# Methicillin‐resistant *Staphylococcus pseudintermedius* (MRSP) from healthy dogs in Norway – occurrence, genotypes and comparison to clinical MRSP

**DOI:** 10.1002/mbo3.258

**Published:** 2015-10-01

**Authors:** Ellen Eide Kjellman, Jannice Schau Slettemeås, Harald Small, Marianne Sunde

**Affiliations:** ^1^ Follo Dyreklinikk AS1400SkiNorway; ^2^ Norwegian Veterinary InstitutePb 750 Sentrum0106OsloNorway

**Keywords:** Antimicrobial resistance, carrier, dog, MRSP

## Abstract

The aim of the study was to investigate the occurrence of methicillin‐resistant *Staphylococcus pseudintermedius* (MRSP) in healthy dogs and further to determine genetic relatedness between carrier isolates and clinical MRSP from dogs in Norway. A total of 189 healthy dogs visiting ten veterinary clinics were screened for MRSP during the period February to April 2013. Carrier isolates were susceptibility tested with disk diffusion and genotyped using multilocus sequence typing (MLST) and pulsed‐field gel electrophoresis (PFGE). Forty‐nine clinical MRSP were characterized for comparison. These isolates were collected from July 2008 to April 2013 and represent all MRSP index isolates from each MRSP‐positive dog detected in Norway until April 2013. Geographical distribution of all MRSP cases was investigated using the ArcGIS 9.3 Software. MRSP was detected from five (2.6%) healthy dogs, sampled at three different clinics. The isolates grouped into three sequence types (STs): ST252 (two isolates), ST71 (two isolates) and ST306 (one isolate). MRSP from dogs sampled at the same animal clinic belonged to the same ST and produced identical PFGE pattern. The 49 clinical MRSP grouped into 15 STs; ST258 (*n* = 17), ST71 (*n* = 10), and ST305 (*n* = 4) were the most prevalent. The MRSP carrier isolates were genetically related to MRSP variants from dogs with infections as ST306 (from a carrier) is related to ST258. MRSP ST252, found in two carriers, was also present among the clinical MRSP isolates. Altogether the MRSP isolates were genetically diverse and MRSP of other lineages than ST71 continues to disseminate in Norway. Susceptibility testing showed that MRSP isolates of the ST71 lineage were the most multiresistant. Our study showed that MRSP could be detected in healthy dogs without infections and with no recent history of antimicrobial therapy stressing the need for future monitoring, infection control and prudent use of antimicrobial agents.

## Introduction

*Staphylococcus pseudintermedius* is considered a major pathogen in dogs, typically involved in skin and ear infections, urinary tract infections, and postoperative infections. *Staphylococcus pseudintermedius* is also commonly a part of the normal flora of healthy dogs. Several studies have found the carrier prevalence in healthy dogs to be as high as 69% (Paul et al. 2012) and 87.4% (Rubin and Chirino‐Trejo 2011). The zoonotic potential is not as striking as that of *Staphylococcus aureus* but the organism has been isolated both from healthy owners with infected pets and from veterinarians working at small animal clinics (Guardabassi et al. 2004; Anonymus 2009; van Duijkeren et al. 2011). *Staphylococcus pseudintermedius* has also been responsible for severe bacterial infections in humans (Anonymus 2009; Stegmann et al. 2010; Riegel et al. 2011).

Methicillin‐resistant variants of *S. pseudintermedius* (MRSP) are of growing concern in small animal veterinary medicine. The emergence of MRSP in dogs represents a relatively new problem with the first single observation made in 1999 from a dog in the US (Gortel et al. 1999). In Europe, MRSP quickly emerged after the first findings in 2005–2006 (Loeffler et al. 2007; Schwarz et al. 2008; Ruscher et al. 2009, 2010). MRSP have a clonal distribution with MRSP belonging to sequence type (ST)71 as the dominating lineage in Europe and ST68 as the main lineage in the United States (Black et al. 2009; Perreten et al. 2010). This is in contrast to the methicillin‐susceptible *S. pseudintermedius* population (MSSP) which is associated with high genetic diversity (Norstrom et al. 2009). The success of MRSP belonging to ST71 is noteworthy. Recently, MRSP ST71 was described from a dog in South America (Quitoco et al. 2013) documenting its spread to new areas. A previous study from Norway has demonstrated clinical MRSP from dogs to be more genetically diverse than elsewhere (Osland et al. 2012). This study investigated a limited number of isolates, as only isolates from the first 23 MRSP cases in Norway were included. However, clonal spread of non‐ST71 MRSP variants was demonstrated (Osland et al. 2012).

Healthy nonsymptomatic carriers of MRSP may act as reservoir for the microbe and contribute to further spread to other dogs. The carriers may also introduce MRSP to the environment flora of kennels and animals clinics. As companion animals live in close contact with their owners and handlers, zoonotic transmission of MRSP to humans cannot be excluded. Knowledge about MRSP carriage in the healthy dog population is therefore of importance.

The current knowledge about MRSP carrier prevalence among healthy dogs is limited. Most studies have included both healthy dogs and dogs with clinical symptoms like pyoderma and ear infections. The prevalence of MRSP carriage in healthy dogs has been found to vary; from none (Murphy et al. 2009; Gharsa et al. 2013; Wedley et al. 2014), to 1.6% (Mouney et al. 2015) and 4.6%. (Gomez‐Sanz et al. 2011). The MRSP recovery rate seems to be higher when both diseased and healthy dogs are screened (Hanselman et al. 2008; Nienhoff et al. 2011; Feng et al. 2012).

In humans, *S. aureus* colonization is a predisposing risk factor for infection (von Eiff et al. 2001). There are knowledge gaps concerning *S. pseudintermedius* carriage and infection risks in dogs, but it is reasonable to believe that persistent carriers may be at higher risk of getting infection caused by *S. pseudintermedius* than noncarriers.

The aim of the study was to investigate the MRSP occurrence among healthy dogs with no recent history of antimicrobial therapy visiting veterinary clinics in a defined area in Norway. Furthermore, genetic relationship between carrier isolates and MRSP from infected dogs in Norway was investigated.

## Material and Methods

### Collection of samples from healthy dogs

Ten small animal clinics in Oslo and the surrounding counties were invited to participate in the study. Our initial sampling plan included 200 healthy dogs coming in for routine examination or treatment. A written instruction was sent out to the participating clinics in advance. Dogs included should be healthy, not treated with antimicrobial agents during the last 14 days before sampling (in case of cefovecin treatment it should be more than 64 days). They should not have any infected wounds, ear inflammation, pyoderma, furunculosis, or other on‐going infections. Dogs with a history of skin disorders (like atopic dermatitis) should not be included in the study. The sampling was carried out from February to April 2013. One sterile swab was used to sample the mouth and the perineum of each dog. The swab was placed in a coal transport medium, labeled, and sent to the laboratory the same day or the following day (after storage at 4°C). All the required material was supplied to the clinics and included swabs, forms (where the clinic were to fill in the dog breed, age, sex, and owner's zip code), and envelopes with prepaid postage. Participation in the study and sampling of dogs were performed on a voluntary basis. We received samples from 189 dogs attending nine small animal clinics.

### Clinical MRSP isolates

A total of 49 MRSP isolates from 49 dogs with infections at different body sites were investigated further. These isolates were collected from July 2008 to April 2013 and represent all MRSP index isolates from each MRSP‐positive dog detected in Norway until April 2013. The isolates originated from clinical samples received at our diagnostic service at the Norwegian Veterinary Institute (NVI). This laboratory is the reference laboratory for antimicrobial resistance in the veterinary sector in Norway and receives all MRSP isolates detected in four connected laboratories localized in other regions of the country. The collection of MRSP originates from dogs from all parts of the country. Species identification and *mecA* detection has been carried out by the use of previously described PCR methods. Different PCR methods have been used over the years for this purpose (Predari et al. 1991; Poulsen et al. 2003; Bannoehr et al. 2009; Sasaki et al. 2010; Stegger et al. 2012).

### Isolation and verification of MRSP from healthy dogs

Detection of MRSP from healthy dogs was carried out using the following procedure: The swab was placed in 9 mL Mueller Hinton broth (Difco, Becton Dickinson and Company, Sparks, MD) with 6.5% NaCl and incubated overnight at 37°C. Subsequently 10 *μ*L of the overnight culture was plated out on Brilliance MRSA 2 agar (Oxoid, Oslo, Norway) and incubated at 37°C for 24 h and for 48 h. Presumptive MRSP isolates were selected and sub‐cultured on blood agar. PCR was used for species identification (Sasaki et al. 2010) and *mecA/mecC* investigation (Poulsen et al. 2003; Stegger et al. 2012). Controls used were: *S. pseudintermedius* CCUG 49543 (*mec* ^−^), *S. aureus* CCUG 35603 (*mecA* ^+^), *S. aureus* ab773 (*mecC* ^+^), *S. aureus* CCUG 29213 (*mec* ^−^).

### Susceptibility testing of MRSP isolates

All clinical and carrier MRSP isolates were subjected to susceptibility testing by the use of the disk diffusion methodology recommended by the European Committee on Antimicrobial Susceptibility Testing (www.eucast.org). The following antimicrobial agents were included in the test panel: Tetracycline, fusidic acid, trimethoprim/sulfamethoxazole, ciprofloxacin, erythromycin, clindamycin, gentamicin, nitrofurantoin, penicillin, ampicillin, amoxicillin/clavulanate, and cefoxitin. *Staphylococcus aureus* ATCC 29213, and MRSA CCUG 35603 were included as quality controls. Categorization of the isolates as resistant or susceptible was based on clinical breakpoints recommended for *S. aureus* by EUCAST. For cefoxitin a recently recommended breakpoint for *S. pseudintermedius* was used; *S* ≥ 35, *R* < 35 (www.eucast.org). All isolates were classified as resistant to beta‐lactam antimicrobial agents based on *mecA* presence.

### Genotyping

All MRSP isolates were subjected to multilocus sequence typing (MLST) based on a seven locus technique (Solyman et al. 2013). After PCR amplification the sequences of the amplicons were determined by the use of BigDye Terminator v3.1/1.1 Cycle Sequencing kit (Applied Biosystems, Foster City, CA). The sequencing reactions were run on a capillary sequencer (3130*xl* Genetic Analyzer, Applied Biosystems). Resulting sequences were analyzed using CLC Main Workbench 6 (CLC bio a Qiagen Company, Aarhus, Denmark) and the ST numbers were allocated by using the pubMLST website for *S. pseudintermedius*. Phylogenetic analysis was performed by the use of the MEGA 6 software with neighbor‐joining method using maximum composite likelihood method (www.megasoftware.net). The total length of the alignment was 2944 characters and consisted of the *tuf*,* cpn60*,* pta*,* purA*,* fdh*,* sar*,* ack* gene sequences. MRSP from healthy dogs were subjected to pulsed‐field gel electrophoresis (PFGE) using a protocol described previously (Norstrom et al. 2009). Banding patterns were evaluated by visual inspection and by the use of BioNumerics software (BioNumerics, Applied Maths, Kortrijk, Belgium).

### Geographical information and statistical methods

Handling of geographical data and generation of maps were performed using the ArcGIS 9.3 Software (ESRI, Redlands, CA). Statistical differences were calculated using chi‐square test.

## Results

### MRSP from healthy dogs

MRSP was isolated from five (2.6%) of the 189 healthy dogs. Positive dogs were sampled at three clinics located in geographically distinct areas. From two clinics, two MRSP‐positive dogs were detected. None of the MRSP‐positive dogs belonged to the same household. The mean age of positive dogs was 6.9 years, ranging from 1.25 to 9 years. The majority of MRSP carriers were older, from 7 to 9 years, only one was younger than 2 years. The gender distribution of the positive dogs was equal, with two males and three females. The dogs were of five different breeds; Labrador retriever, Miniature Pinscher, Border terrier, German shepherd, and Bichon frisé.

### Genotyping of MRSP isolates

MLST showed that the MRSP carrier isolates belonged to three different STs: ST306 (one isolate), ST71 (two isolates), and ST252 (two isolates). ST306 is related to ST258 and ST261, these STs were also found in our collection of clinical MRSP isolates as described below. Carrier isolates of the same ST originated from dogs sampled at the same clinic. PFGE of MRSP from dogs sampled at the same clinic produced identical banding patterns when pairwise compared, indicating a possible common source.

The 49 clinical MRSP isolates grouped into 15 different STs. MRSP isolates belonging to ST258 occurred most frequently (*n* = 17), followed by ST71 (*n* = 10), ST305 (*n* = 4), and ST261, ST298, ST299 (all with three isolates). The remaining nine isolates belonged to nine different STs; ST41, ST180, ST252, ST300, ST301, ST302, ST303, ST304, ST307. Isolates belonging to ST261 (*n* = 3) are related to ST258 (sequence difference in one allele), in the same way are MRSP ST180 (*n* = 1) and MRSP ST304 (*n* = 1) related to ST71 and ST305, respectively (sequence differences in one allele).

**Figure 1:**
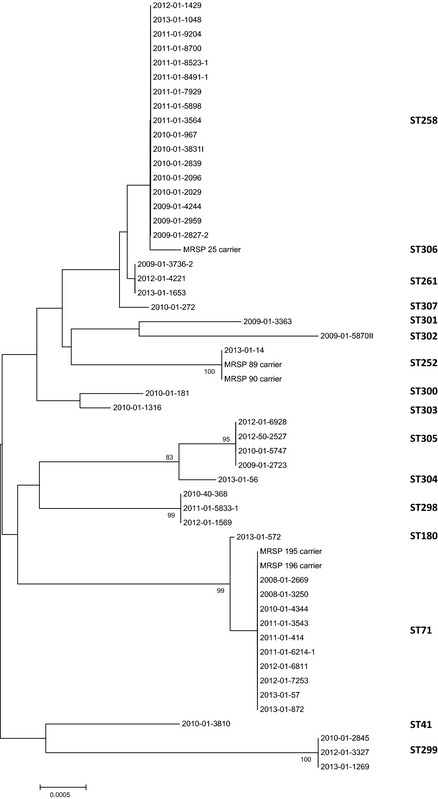
Neighbor‐joining and bootstrapping analysis of concatenated sequence alignment (*tuf*,* cpn60*,* pta*,* purA*,* fdh*,* sar*,* ack*). Five‐hundred bootstrap replicates were used to determine the confidence of the tree. Only bootstrap values larger than 75 are shown.

In a previous study we have typed the 23 first MRSP from dogs in Norway with a former five locus based MLST method (Osland et al. 2012). All isolates typed as ST71 (*n* = 4) by the former method were still ST71 by the use of the novel seven locus technique. All isolates previously typed as ST106 (*n* = 8) belonged to ST258 in this study, two isolates typed as ST127 belonged to ST305 by the use of the novel MLST, and finally two ST28 isolates, were typed to two different STs (303 and 307) with the new method. A phylogenetic tree, with concatenated sequence data from all clinical and carrier isolates investigated in this study, is shown in Figure 1.

### Resistance to antimicrobial agents

Resistance profiles of all 54 MRSP (carrier and clinical) isolates are shown in Table 1. The resistance properties associated with the various STs varied: The isolates belonging to the ST71 lineage were the most resistant, expressing resistance to five to seven of the eight substances listed in Table 1. Resistance to ciprofloxacin and gentamicin occurred significantly more often among isolates of the MRSP ST71 lineage than among non‐ST71 isolates (*P *<* *0.01, chi‐square test). The resistance profiles among isolates grouping within the different STs were relatively stable, with exception of tetracycline and fusidic acid resistance, where some variations occurred (Table 1). Similar resistance properties were found for isolates within STs related to ST258; one ST306 and three ST261. In the same way were the resistance properties of ST180 and ST304, similar to the resistance properties of ST71 and ST305 isolates.

**Table 1:** Reistance to non‐beta‐lactams among 54 MRSP (carrier and clinical isolates) investigated

Isolate number	ST	TMS	TET	FUS	CIP	ERY	CLI	GEN	NIT
2009‐01‐2827	258	R	S	S	S	R	R	S	S
2009‐01‐2959	258	R	R	S	S	R	R	S	S
2009‐01‐4244	258	R	R	S	S	R	R	S	S
2010‐01‐967	258	R	R	S	S	R	R	S	S
2010‐01‐2029	258	R	R	S	S	R	R	S	S
2010‐01‐2096	258	R	R	S	S	R	R	S	S
2010‐01‐2839	258	R	R	S	S	R	R	S	S
2010‐01‐3831	258	R	S	S	S	R	R	S	S
2011‐01‐3564	258	R	R	S	S	R	R	S	S
2011‐01‐5898	258	R	R	S	S	R	R	S	S
2011‐01‐7929	258	R	S	S	S	R	R	S	S
2011‐01‐8491	258	R	S	S	S	R	R	S	S
2011‐01‐8523	258	R	S	S	S	R	R	S	S
2011‐01‐8700	258	R	R	S	S	R	R	S	S
2011‐01‐9204	258	R	R	S	S	R	R	S	S
2012‐01‐1429	258	R	S	S	S	R	R	S	S
2013‐01‐1048	258	R	R	S	S	R	R	S	S
2009‐01‐3736	261	R	R	S	S	R	R	S	S
2012‐01‐4221	261	R	R	R	S	R	R	S	S
2013‐01‐1653	261	R	R	S	S	R	R	S	S
MRSP 25 carrier	306	R	S	S	S	R	R	S	S
2008‐01‐2669	71	R	S	R	R	R	R	R	S
2008‐01‐3250	71	R	S	R	R	R	R	R	S
2010‐01‐4344	71	R	S	S	R	R	R	R	S
2011‐01‐414	71	R	S	S	R	R	R	R	S
2011‐01‐3543	71	R	R	S	R	R	R	R	S
2011‐01‐6214	71	R	S	S	R	R	R	R	S
2012‐01‐6811	71	R	S	S	R	R	R	R	S
2012‐01‐7253	71	R	R	R	R	R	R	R	S
2013‐01‐57	71	R	S	S	R	R	R	R	S
2013‐01‐872	71	R	R	S	R	R	R	R	S
MRSP 195 carrier	71	R	S	S	R	R	R	R	S
MRSP 196 carrier	71	R	S	S	R	R	R	R	S
2013‐01‐572	180	R	R	S	R	R	R	R	S
2009‐01‐2723	305	S	S	R	S	S	S	S	S
2010‐01‐5747	305	S	S	R	S	S	S	S	S
2012‐01‐6928	305	S	S	R	S	S	S	S	S
2012‐50‐2527	305	S	S	R	S	S	S	S	S
2013‐01‐56	304	S	S	R	S	S	S	S	S
2010‐40‐368	298	R	R	S	S	R	R	R	S
2011‐01‐5833	298	R	R	S	S	R	R	R	S
2012‐01‐1569	298	R	R	R	S	R	R	R	S
2010‐01‐2845	299	S	S	S	S	R	R	S	S
2012‐01‐3327	299	S	S	S	S	R	R	S	S
2013‐01‐1269	299	S	S	S	S	R	R	S	S
2013‐01‐14	252	S	S	R	S	R	R	R	S
MRSP 89 carrier	252	S	S	R	S	R	R	R	S
MRSP 80 carrier	252	S	S	R	S	R	R	R	S
2010‐01‐3810	41	S	S	R	S	R	R	S	S
2010‐01‐181	300	R	R	S	S	R	R	R	S
2009‐01‐3363	301	R	R	S	S	R	R	R	S
2009‐01‐5870II	302	R	S	S	S	R	R	S	S
2010‐01‐1316	303	R	R	S	S	S	S	S	S
2010‐01‐272	307	R	R	S	S	S	S	S	S

MRSP, methicillin‐resistant *Staphylococcus pseudintermedius*; ST, sequence type; TMS, trimethoprim/sulfamethoxazole; TET, tetracycline; FUS, fusidic acid; CIP, ciprofloxacin; ERY, erythromycin; CLI, clindamycin; GEN, gentamicin; NIT, nitrofurantoin; S, susceptible; R, resistant.

### Geographical distribution of MRSP

**Figure 2:**
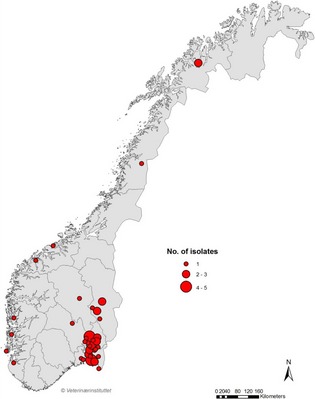
Geographical distribution (based on owner's home address) of all (*n* = 54, clinical and carrier) methicillin‐resistant *Staphylococcus pseudintermedius* isolates.

**Figure 3:**
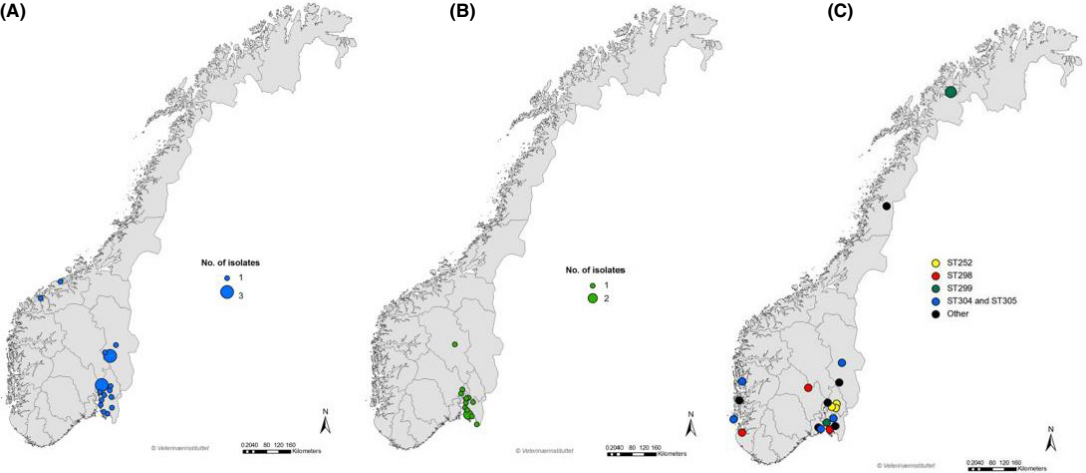
Geographical distribution of the most common methicillin‐resistant *Staphylococcus pseudintermedius* sequence types (STs) in Norway (clinical and carrier isolates). (A) ST258 (and the related ST261 and ST306), (B) ST71 (and the related ST108), and (C) remaining MRSP variants: ST252, ST298, ST299, ST305/304. The group called “other” comprise single isolates belonging to: ST41, ST300, ST301, ST302, ST303, and ST307.

The geographical distribution of MRSP in Norway (based on home address of the owner) is shown in Figure 2, and the distribution of the most prevalent STs in Figure 3A and B. Clinical MRSP originated from all parts of the country, however, there was a clustering around the capital Oslo and in the southern and eastern part of Norway, corresponding to the most densely populated area in Norway (resulting in a higher number of samples from this region). Geographic clustering of a specific ST was observed for three ST252; two carrier isolates and one clinical isolate (Fig. 3C). In addition, a few ST258 and a few ST71 were identified within the same geographical area (Fig. 3A and B).

## Discussion

MRSP was isolated from 2.6% of the healthy dogs included in this study. The MRSP reservoir in healthy individuals is worrisome since carriers are nonsymptomatic and could easily contribute to further MRSP dissemination. The size of this study was relatively small, and it should therefore be considered as a pilot‐study. However, as we detected five carriers it is possible that MRSP circulates among healthy dogs in Norway. This should be further investigated in new prevalence studies including a higher number of sampled dogs.

In a recent study from the UK, sampling 724 dogs, no MRSP carriers were found (Wedley et al. 2014). This may indicate differences in the geographical distribution of MRSP. However, differences in sampling and laboratory methods may affect the recovery rate. We sampled the mouth and perineal region as these sites came out as the most optimal sites in a previous, although limited, study (Paul et al. 2011).

The MRSP‐positive healthy dogs may be transient carriers or only contaminated with MRSP, instead of truly colonized. Studies including follow‐up sampling of positive individuals should be performed in order to get more knowledge about the persistence of carriage in healthy dogs.

Among the MRSP carriers five different breeds were represented. A genetic breed predisposition for atopic skin disease is described for German shepherd (Scott and Paradis 1990) and Labrador retriever (Sture et al. 1995; Willemse 1996), but as we sampled healthy dogs only this is of little relevance for the carrier status of the investigated dogs. One study has shown that the adherence of *S*. (*pseud*)*intermedius* to the corneocytes of atopic dogs is significantly greater than to those of healthy dogs and that adherence is significantly greater in dogs with high levels of pruritus compared to those with low scores of pruritus (Simou et al. 2005). It is therefore possible that dogs with atopic skin disease have a higher risk of becoming MRSP carriers. Another study found that the use of antimicrobials in dogs with non‐MRSP infection increased the risk of finding MRSP after treatment (Beck et al. 2012). A study from Germany has shown that dogs that had been hospitalized, had frequent visits to clinics, received topical ear medication, or glucocorticoids were at higher risk of MRSP infections (Lehner et al. 2014). Surgical intervention may also be a significant risk factor (Anonymus 2009). A recent study from Finland describing a large MRSP outbreak found that skin lesions, antimicrobial therapy and prolonged hospitalization were risk factors for MRSP (Gronthal et al. 2014). The same study also described the use of a “search‐and‐isolate” strategy, including screening of the risk patients, for control of MRSP (Gronthal et al. 2014).

In this study, healthy dogs sampled at the same clinic carried MRSP of the same genotype. It is possible that the dogs were contaminated with MRSP in connection with the visit at the clinic, possibly via environmental flora and/or direct dog contact. Another explanation could be that the particular MRSP variant(s) may circulate among individuals of the local dog population. Among the clinical isolates investigated in this study one MRSP ST252 was identified. A two band difference in PFGE pattern was observed between the pattern of this isolate and the ST252 carrier isolates (data not shown). The three isolates were recovered from dogs belonging to different households but living within the same geographical area (Fig. 3C). The clinical isolate originated from a submission received in January 2013, whereas the carrier isolates were detected 1 month later. The dogs were sampled at different clinics. These findings may indicate a local distribution of this MRSP variant.

From one healthy dog MRSP ST306 was isolated. This ST is related to ST258, the most common ST among the clinical MRSP isolates with one nucleotide difference in the *pta* gene. The other two MRSP variants recovered from healthy dogs, MRSP ST71, and ST252, were also represented in our collection of clinical MRSP isolates. To our knowledge this is the first report of canine MRSP carrier and clinical isolates recovered from dogs in the same geographical area. A study on human MRSA has shown that carrier and clinical isolates from the same area often belonged to the same genotype (Bae et al. 2010). However, further studies including a higher number of carrier isolates are required in order to investigate if there are clonal differences among colonizing and clinical MRSP isolates in dogs.

The first two cases of canine MRSP infection in Norway, dating back to 2008, involved MRSP ST71. These isolates generated PFGE banding patterns with a high degree of similarity to the major European MRSP clone ST71‐t02‐SCC_mec_ II‐III (Osland et al. 2012). Most of the subsequent MRSP ST71 detected in Norway exhibit a similar banding pattern (data not shown). In a previous study we also showed that isolates belonging to ST258 (formerly typed to ST106) had a rather conserved PFGE pattern (Osland et al. 2012). Similar PFGE patterns were also generated with DNA from most of more recent ST258 isolates also including the related ST306 from a healthy carrier (data not shown).

The MRSP isolates investigated, including carrier and clinical isolates belonged to a variety of STs. There is a continuous spread of MRSP variants belonging to other lineages than ST71 in Norway, such as MRSP ST258. Genetic diversity among clinical MRSP has recently been reported from Denmark, also comprising isolates belonging to the ST258 lineage (Damborg et al. 2013). MRSP ST258 may represent an emerging MRSP clone in Northern Europe and a shift to a more polyclonal MRSP population may be underway. The genetic diversity of MRSP isolates recovered from dogs in Norway is noteworthy. There is no obvious reason for this but differences in climate, dog population and dog density may affect the bacterial population. Import and travel abroad with pets may also be a contributing factor. Detection of MRSP in the primary diagnostic may also be challenging; it is a general perception that MRSP has a multiresistant nature. However, our collection of MRSP contains isolates expressing resistance to beta‐lactam and fusidic acid only.

More knowledge about possible ways of eradication of MRSP from dogs is highly demanded. Some studies suggest that carriage can persist for a long time after detection and treatment of MRSP pyoderma, surgical site infection, and infection after traumatic injuries (Beck et al. 2012; Windahl et al. 2012). There are currently no scientifically based guidelines regarding how to eradicate MRSP from healthy dogs. The rapid rise of MRSP as a pathogen in veterinary small animal practices is of great concern. Not just as a threat to animal health, but also as a potential zoonosis. It is difficult to envision a possible way to stop MRSP from spreading between countries and between veterinary clinics, as there are few restrictions on traveling with pets, and as MRSP has been shown to be easily transferred between dogs. Prudent use of antimicrobial agents to companion animals is important for control of MRSP. In addition, there are guidelines available for how veterinary clinics should prevent contamination of the clinic, and minimize the risk of transferring MRSP to other patients (Weese 2012). It is increasingly important to implement these in order to control emerging pathogens, like MRSP, in small animal veterinary medicine.

## Conflict of Interest

None declared.
